# Transcriptomic and Metabolomic Analysis of Seedling-Stage Soybean Responses to PEG-Simulated Drought Stress

**DOI:** 10.3390/ijms23126869

**Published:** 2022-06-20

**Authors:** Xiyue Wang, Shuang Song, Xin Wang, Jun Liu, Shoukun Dong

**Affiliations:** 1 College of Agriculture, Northeast Agricultural University, Harbin 150030, China; xiyuewang2021@163.com (X.W.); ss2362351835@126.com (S.S.); w324ase@163.com (X.W.); 2 Lab of Functional Genomics and Bioinformatics, Institute of Crop Science, Chinese Academy of Agricultural Sciences, Beijing 100081, China; liujun@caas.cn

**Keywords:** soybean, drought stress, seedling stage, omics analysis

## Abstract

Soybean is an important crop grown worldwide, and drought stress seriously affects the yield and quality of soybean. Therefore, it is necessary to elucidate the molecular mechanisms underlying soybean resistance to drought stress. In this study, RNA-seq technology and ultra-performance liquid chromatography–tandem mass spectrometry were used to analyze the transcriptome and metabolome changes in soybean leaves at the seedling stage under drought stress. The results showed that there were 4790 and 3483 DEGs (differentially expressed genes) and 156 and 124 DAMs (differentially expressed metabolites), respectively, in the HN65CK vs. HN65S0 and HN44CK vs. HN44S0 comparison groups. Comprehensive analysis of transcriptomic and metabolomic data reveals metabolic regulation of seedling soybean in response to drought stress. Some candidate genes such as *LOC100802571*, *LOC100814585*, *LOC100777350* and *LOC100787920*, *LOC100800547*, and *LOC100785313* showed different expression trends between the two cultivars, which may cause differences in drought resistance. Secondly, a large number of flavonoids were identified, and the expression of Monohydroxy-trimethoxyflavone-O-(6″-malonyl)glucoside was upregulated between the two varieties. Finally, several key candidate genes and metabolites involved in isoflavone biosynthesis and the TCA cycle were identified, suggesting that these metabolic pathways play important roles in soybean response to drought. Our study deepens the understanding of soybean drought resistance mechanisms and provides references for soybean drought resistance breeding.

## 1. Introduction

Humans have domesticated and enhanced many crop types over thousands of years of agricultural growth, making them the basis of our daily diet, animal feed, and industrial applications [1]. Soybeans [*Glycine max* (Linn.) Merr.] are the most widely grown legumes in the world and are a great source of protein and oil. The main soybean varieties in production usually have nutritional characteristics of high protein, high fat, low carbohydrate, high dietary fiber, and extensive processing characteristics [2]. Global soybean and hectare yields have gradually increased over the last century, but to fulfill the growing demand of the world population, yields must increase at a faster rate than they are now [3]. In recent years, a large number of germplasm resources have been developed owing to advancements in breeding technology. To adjust to market demand, support sustainable agricultural development, and respond to future environmental changes, soybean breeding must be hastened [4]. Abiotic and biotic stresses (such as pests and diseases, high temperatures, drought, salt, flooding, and cold) are common in soybean production because of various environmental conditions. These factors cause yield loss, lowering total soybean output [5].

Among these abiotic stresses, drought is a major limiting factor for soybean production. Drought alone causes greater crop yield losses than all pathogens combined [6]. Soybeans are regarded as one of the most drought-sensitive crops, with output losses of up to 40% in the worst years [7]. Drought has a series of effects on plants from morphology to physiology, including changing leaf or root morphology, stimulating osmotic regulation systems, enhancing reactive oxygen species metabolism and hormone metabolism, and reducing photosynthesis [8]. Under drought stress, the contents of abscisic acid (ABA), auxin (AUX), ethylene (ET), salicylic acid (SA), brassinolide (BR), and jasmonic acid (JA) may change significantly to confer plants some drought resistance [9]. In addition, antioxidant and osmotic regulation systems are also activated, mainly manifested as the enhancement of antioxidant enzyme activity, the accumulation of soluble sugar and soluble protein, and an increase in proline content [10]. However, changes at the physiological level cannot fully explain the complex mechanism of plant drought resistance, and the synthesis of these enzymes or hormones is usually mediated by certain genes or small molecule metabolites. Some genes and metabolites have been shown to play a role in drought regulation. For example, isoflavones have strong antioxidant capabilities and can protect DNA from free radical damage. Genistein and daidzein are two significant and efficient antioxidants found in soy isoflavones [11]. Second, some transcription factors have been confirmed to be the main players in water stress signals, including MYB DREBs, NAC, bZIP, AP2/ERF, and AREB/ABF, which respond to drought by affecting stomatal movement or certain metabolic pathways [12,13,14]. Heat shock protein (HSP), thioredoxin, ascorbic acid, glutathione, and hydrogen peroxide (H_2_O_2_)-related proteins have been shown to be upregulated or downregulated in drought-treated tobacco leaves at the protein level [15]. However, the effects of plant drought are extremely complex and further research is needed to uncover more drought-affected genes, proteins, and metabolites and use them for breeding improvement.

At present, in the study of plant stress resistance, selecting varieties with different stress resistances and conducting research at the same time can make the results more convincing and reveal the reasons for the differences in stress resistance among varieties. Using comparative proteomics analysis, researchers discovered that peroxidase, MDH, and SAMS were greatly enhanced in the roots or leaves of SE (drought-resistant genotype) and dramatically decreased in SW (drought-sensitive genotype) in response to drought stress [16]. Transcriptome and metabolomic studies were conducted on the responses of *Lycium chinese* (LC) and *Lycium ruthenicum* (LR) to salt stress. MAPK signaling and plant hormone signal transduction pathways were over-represented in wolfberry salt response genes. However, LR contains higher flavonoid and flavonoid concentrations than LC [17]. Cultivars with improved abiotic and biotic stress tolerance, increased nutritional value, and agronomic importance must be developed. In general, omics technology, which includes genomics, transcriptomics, proteomics, and metabolomics, is utilized for further molecular research, allowing for a better understanding of the gene, protein, and metabolite activities in plant responses to abiotic stress. These methods are critical for identifying crucial genes, proteins, and metabolic pathways that underpin many important agronomic features and for marker-assisted crop breeding [18].

Therefore, low-water potential treatments are required to study plant responses to drought. However, for soil drought, it is not only to dehydrate the cells of the plant but also to dehydrate the cell walls. Therefore, an osmotic substance that simulates soil drought must have a molecular weight large enough to not penetrate the cell wall and produce the same dehydration effect as that of soil drought. If an osmolyte penetrates the cell wall, enters the cell through the cell membrane, and is metabolized or has other effects, the research problem will be complicated. PEG is an ethanol polymer with a molecular formula of HOCH_2_-[CH_2_-O-CH_2_]n-CH_2_OH and a molecular weight ranging from 200 to 20,000. Experiments have shown that PEG with a molecular weight of 6000 and above cannot penetrate the cell wall, and high molecular weight PEG is an ideal water potential regulator for simulating soil drought [19]. At present, some studies have demonstrated the drought resistance mechanism of soybean using omics technology, but most of them are limited to a single variety or the period of yield formation, and there are few studies on the seedling stage [20]. Based on this feature, this study used 15% PEG-6000 to simulate drought, studied the transcriptome and metabolome changes in two soybean varieties with different drought tolerances at the seedling stage, and analyzed the gene expression and metabolite accumulation of the two varieties. The differences in these aspects provide a theoretical basis for the breeding of drought-resistant plants.

## 2. Results

### 2.1. Sequencing Quality Statistics

Twelve cDNA libraries (three biological replicates for each treatment) were generated and sequenced to better understand the response of the two soybean cultivars to drought stress. The results showed that the number of clean reads of each cDNA library was between 57 million and 65 million. A total of 109.26 G clean reads were obtained, and the clean base distribution of each sample was between 8.45–9.99 G. The distribution of the Q20 bases was between 97% and 98%, the content of the Q30 bases was greater than 92.9%, and the content of the GC bases was between 44.53% and 46.03%. The sequencing quality was good. Clean reads were aligned to the reference genome, and the alignment rate of each sample was above 96.5% (Supplementary Table S1).

### 2.2. Differential Gene Screening

**Figure 1:**
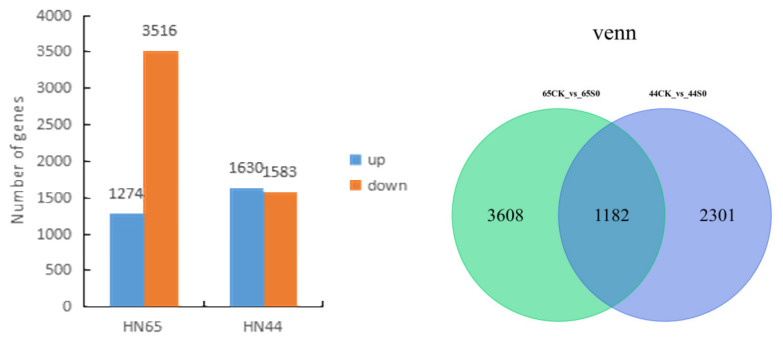
Statistical map of differential genes and VENN map. (**Left**) Statistics of the number of differential genes. (**Right**) Venn diagram of differential genes.

The drought affected the two soybean varieties differently (Figure 1). A total of 4790 differential genes were generated after HN65 drought treatment, including 3516 downregulated and 1274 upregulated genes. A total of 3483 differential genes were generated after HN44 drought treatment, including 1583 downregulated and 1630 upregulated genes. In both cultivars, the number of downregulated genes was greater than that of upregulated genes, and the number of downregulated genes in cultivar HN65 was greater, and drought was more harmful to HN65. A total of 1182 differential genes were found to be co-expressed in the two cultivars by Venn diagram, and HN65 and HN44 responded to drought through their 3608 and 2301 unique genes, respectively.

### 2.3. GO and KEGG Analysis of Differential Genes in Two Varieties

**Figure 2:**
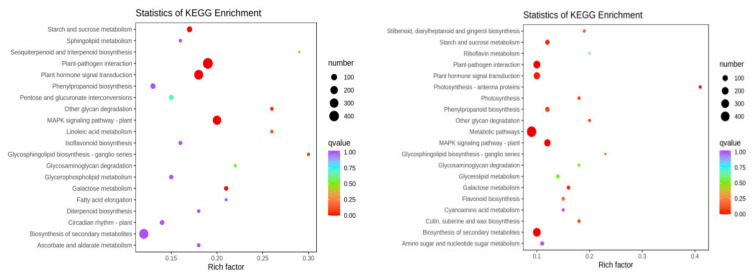
KEGG enrichment bubble map. (**Left**) HN65; (**Right**) HN44.

To clarify the functions of the differentially expressed genes, the differential genes of the two soybean varieties were annotated using GO and KEGG databases (HN65-CK vs. HN65-S0; HN44-CK vs. HN44-S0). PEG-induced upregulated and downregulated genes were annotated into each pathway by GO analysis. In the HN65-CK vs. HN65-S0 group, in the cellular component part, the differential genes were mainly enriched in the plant-type cell wall (7 upregulated, 106 downregulated), anchored component of the membrane (5 upregulated, 102 downregulated), and anchored component of plasma membrane (3 upregulated, 69 downregulated). The differential genes in molecular function were mainly enriched in xyloglucan: xyloglucosyl transferase activity (2 upregulated, 35 downregulated), glucosyltransferase activity (22 upregulated, 77 downregulated), and symporter activity (14 upregulated, 39 downregulated). In the biological process part, the differential genes were mainly enriched in xyloglucan metabolic process (3 upregulated, 40 down-regulated), hemicellulose metabolic process (3 upregulated, 55 downregulated), and cell wall macromolecule metabolic process (4 upregulated, 74 downregulated). In the HN44-CK vs. HN44-S0 group, in the cellular component, the differential genes were mainly enriched in the photosystem (38 downregulated), extracellular region part (23 upregulated, 22 downregulated), and photosystem II (30 downregulated). In the molecular function part, the differential genes were mainly enriched in chlorophyll binding (18 downregulated), pigment binding (11 downregulated), and protein disulfide oxidoreductase activity (8 upregulated, 20 downregulated). In the biological process part, the differential genes were mainly enriched in the hemicellulose metabolic process (15 upregulated, 26 downregulated), cell wall macromolecule metabolic process (17 upregulated, 37 downregulated), and cell wall biogenesis (15 upregulated, 57 downregulated). Only some of the results are shown here, see Supplementary Tables S2 and S3 for details. KEGG enrichment analysis showed (Figure 2) that the differential genes in HN65 were mainly enriched in the plant–pathogen interaction, plant hormone signal transduction, MAPK signaling pathway of the plant, starch and sucrose metabolism, and galactose metabolism. In HN44, differential genes were mainly enriched in the MAPK signaling pathway of the plant, biosynthesis of secondary metabolites, photosynthesis–antenna proteins, metabolic pathways, and plant–pathogen interaction entries. Under drought stress, many pathways were inhibited in both varieties, including photosynthesis, cell wall synthesis, and other pathways.

### 2.4. Gene Expression Differences in Drought-Induced Plant–Pathogen Interaction Pathway and MAPK Signaling Pathway of the Plant

**Figure 3:**
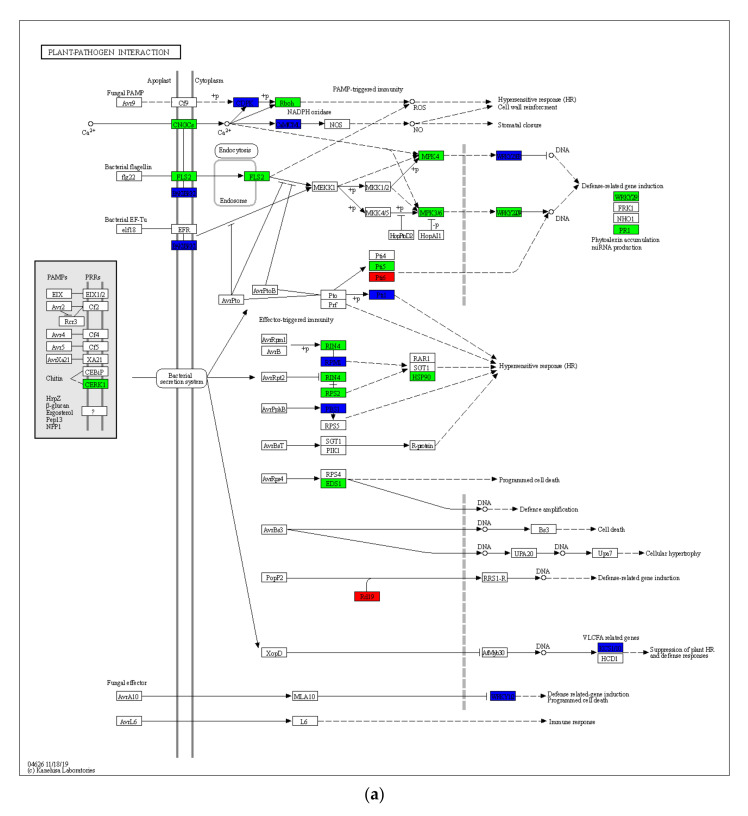

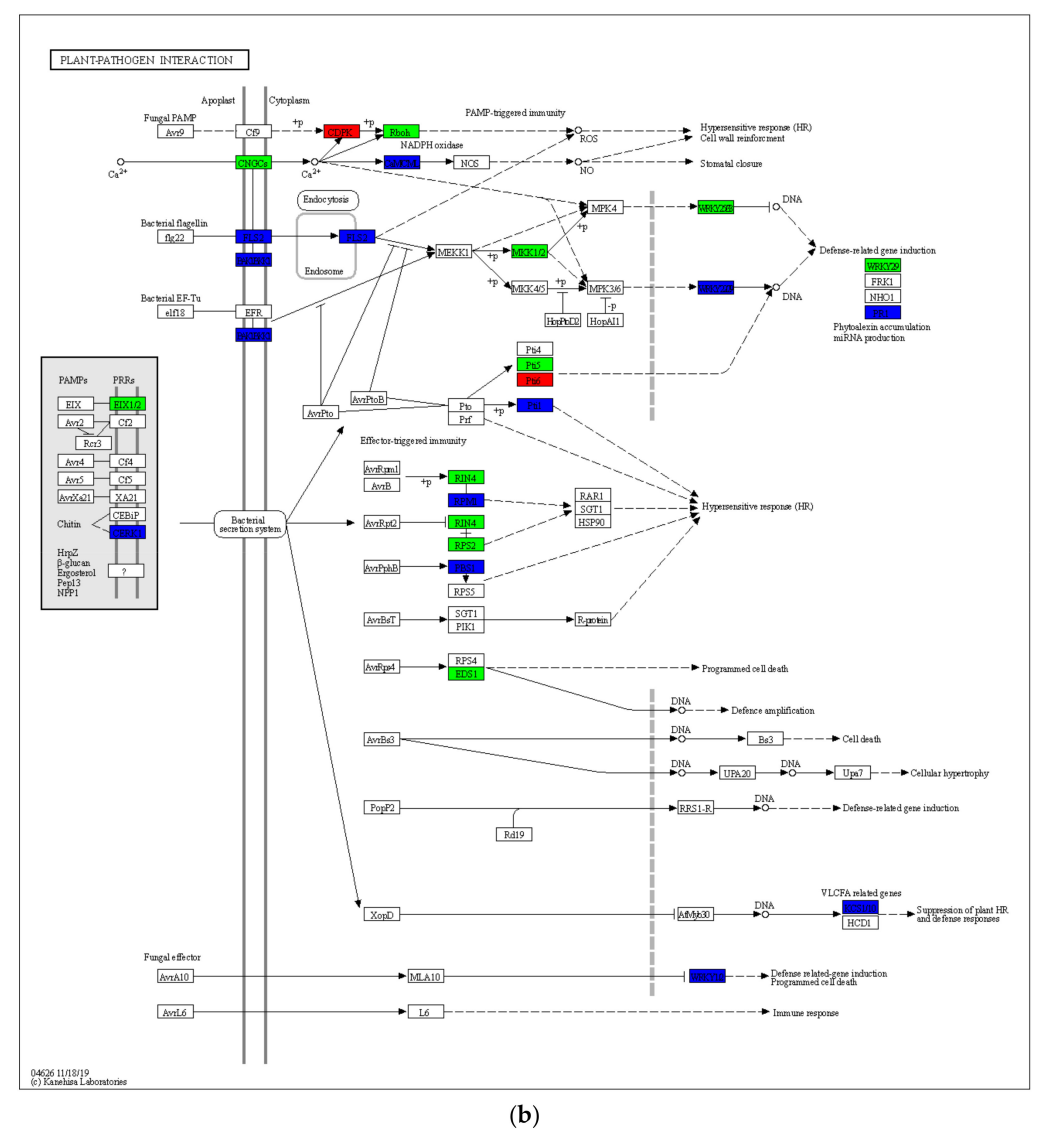
Gene expression of the plant–pathogen interaction pathway. Downregulated genes are represented by green, upregulated genes are represented by red, and both upregulated and downregulated genes are represented by blue, the same below. (**a**) HN65, (**b**) HN44.

Plants have evolved a unique system with numerous levels of resistance to invading pathogens, because they lack animal-like adaptive immune responses. In HN65 (Figure 3), 429 genes were involved in this pathway under drought stress, and most genes were downregulated, with the exception of a few upregulated genes. Chitin elicitor receptor kinase 1 (CERK1) was activated, triggering the plant’s immune response, and all genes in FLS2 and CNGCs related to calcium signaling were downregulated, thereby inhibiting the expression of downstream MAPK-related genes. Furthermore, respiratory burst oxidase (RBOH) was inhibited, thereby reducing reactive oxygen species (ROS) production. A large number of genes related to hypersensitivity were suppressed (mainly genes encoding RIN4, RPM1, RPS2, PBS1, and Pti1), and genes regulating programmed cell death were downregulated (encoding EDS1 and WRKY transcription factor 1) to alleviate drought-induced damage; *LOC100791503*, encoding cathepsin F, was upregulated to induce the expression of plant defense-related genes. In HN44, 222 genes were involved in this pathway under drought stress, chitin elicitor receptor kinase 1 (CERK1) was activated, EXI1/2 expression in PPRs was inhibited, and calcium signaling-related genes in CNGCs all down-regulated, calcium-dependent protein kinase (CDPK) was fully activated (expression was up-regulated) and respiratory burst oxidase (RBOH) was inhibited, thereby reducing ROS production. The genes in FLS2 were partially downregulated, resulting in the repression of downstream MKK1/2 expression, and ultimately the transcription factors WRKY25 and WRKY29, but the upregulation of the gene *LOC100790142* in the pathogenesis-related protein 1 (PR1) may induce the production of antitoxins in plants. Similar to HN65, genes related to hypersensitivity were mostly downregulated, as were the genes that regulate programmed cell death. It is worth noting that the two genes encoding threonine-protein kinase (FLS2) showed opposite expression trends in the two varieties. The log_2_FC of the genes *LOC100802571* and *LOC100814585* were 1.61 and 4.13 in HN44, and −2.11 and −6.26 in HN65, respectively. The different expression patterns of these two genes further affected the expression of related genes in the MAPK pathway, which may be important genes causing differences in drought tolerance between the two cultivars.

**Figure 4:**
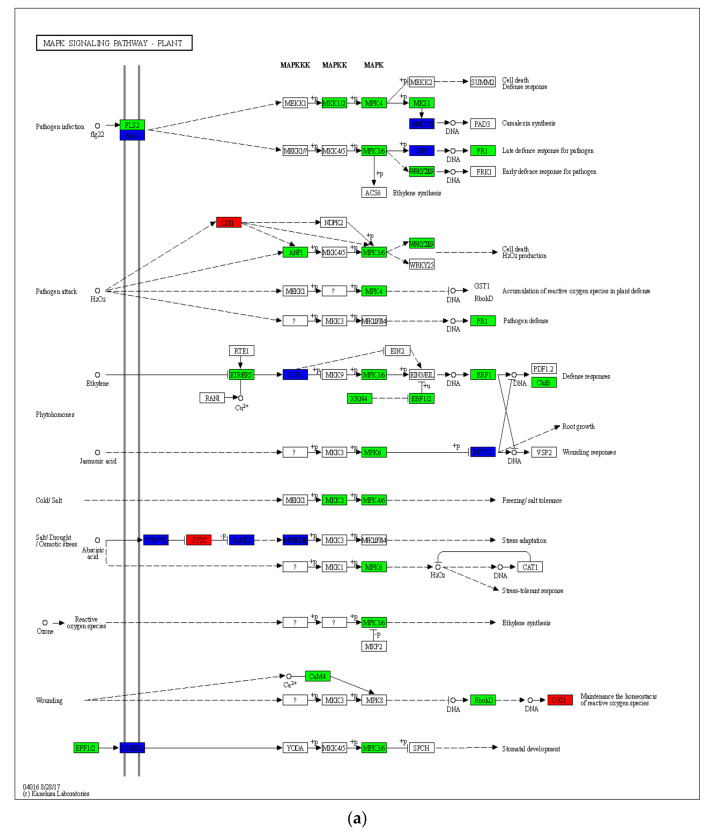

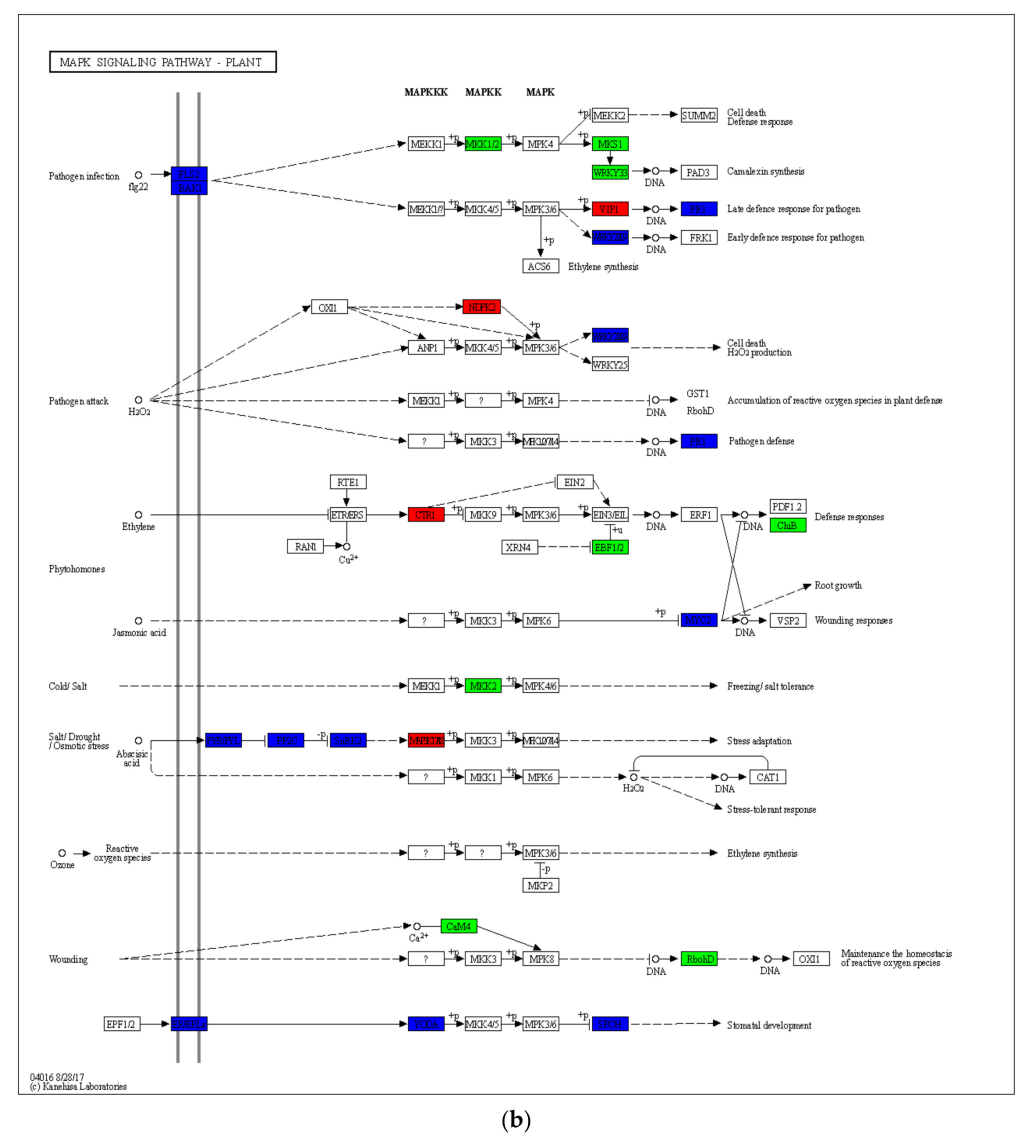
Map of gene expression differences in MAPK signaling pathway of the plant. (**a**) HN65, (**b**) HN44.

The MAPK signaling pathway is activated when FLS2 and EFR are activated, which in turn activates defense genes. Because of the changes in gene expression in FLS2, there were also differences in gene expression in the MAPK pathway between the two types (Figure 4). In HN65, 284 genes were involved in this pathway. MAPKK and MAPK family genes were downregulated (mainly the genes encoding MKK1, MKK2, MPK4, MPK3, MPK6, and MPK4). However, two downstream transcription factors WRKY33 and two genes WRKY36 and BZIP28 in VIP1 were upregulated, the expression of transcription factor WRKY22 was also inhibited, and the defense response of plants to pathogens was inhibited. Stimulated by H_2_O_2_, the expression of genes encoding serine/threonine-protein kinase (OXI1) was upregulated to maintain the balance of reactive oxygen species. However, genes in the MAPK family were downregulated to reduce cell death and H_2_O_2_ production. Plants produce reactive oxygen species under drought stress. Due to the inhibition of MPK3/6 expression, stomatal development was blocked, and ethylene synthesis was reduced. The expression of downstream ethylene-responsive transcription factor 1 (EFR1) was inhibited by ethylene signals, thereby inhibiting plant growth. Plants produce abscisic acid under drought stress; some genes in the abscisic acid receptor PYR/PYL family were upregulated, all genes encoding protein phosphatase 2C were upregulated, and some genes in the MAPKKK family were upregulated, enabling plants to adapt to drought stress. In HN44, changes in the MAPKKK and MAPKK families were mainly involved. The gene *LOC100789241* encoding MKK1/2 was downregulated and the expression of the transcription factor WRKY33 was inhibited, but all the genes encoding the transcription factor VIP1 were upregulated and some genes encoding the transcription factor WRKY22 were upregulated, resulting in the upregulation of the gene *LOC100790142* encoding pathogenesis-related protein 1 (PR1). This may trigger late plant defense responses to pathogens. Under drought stress, some genes encoding abscisic acid receptors in the PYR/PYL family were upregulated, genes encoding protein phosphatase 2C were partly upregulated, and gene *LOC100802174* in the MAPKKK family was upregulated. The overexpression of genes encoding ABA receptors enhances ABA signaling, leading to improved drought tolerance. Unlike HN65, two genes *LOC100777350* (log_2_FC was 2.54) and *LOC100787920* (log_2_FC was 2.15) encoding mitogen-activated protein kinase (YODA) in HN44 were upregulated, and two genes encoding transcription factors SPEECHLESS, *LOC100789928* (log_2_FC was 2.46), and *LOC100784115* (log_2_FC was 4.82), were upregulated to ensure the stomatal development of plants under drought conditions. In general, HN65 was more inhibited in gene expression after drought, with more downregulation, while HN44 was inhibited to a lesser extent and resisted stress through upregulated (or unique pathway) expression of some genes.

### 2.5. Drought-Induced Differences in Plant Hormone Signaling and Other Pathways

**Figure 5:**
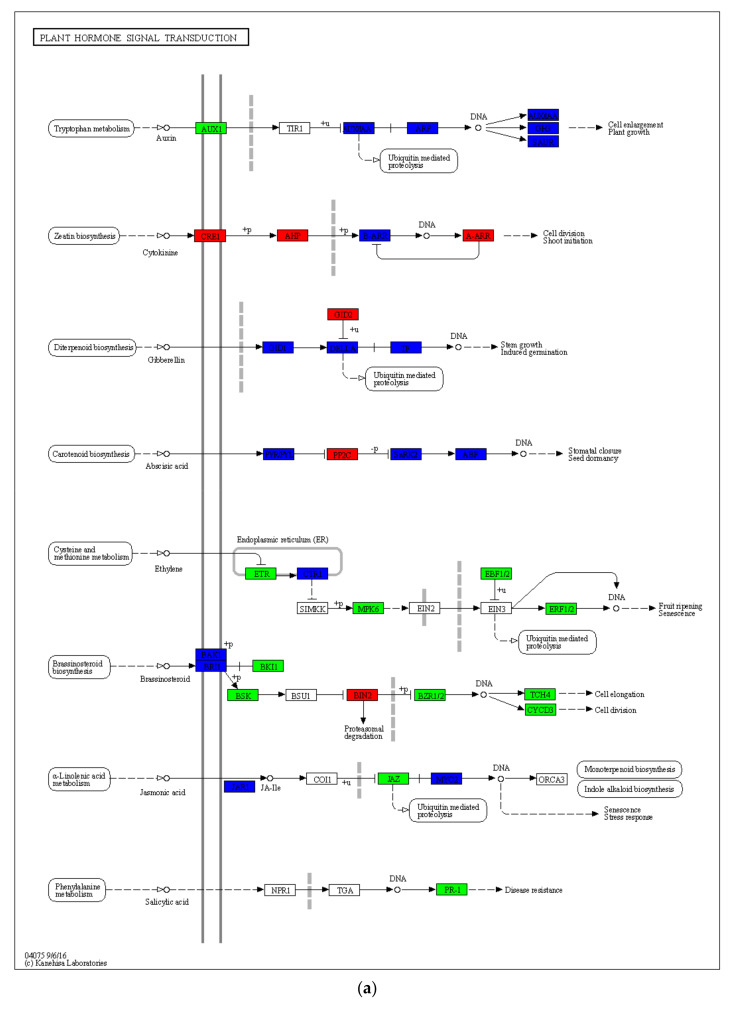

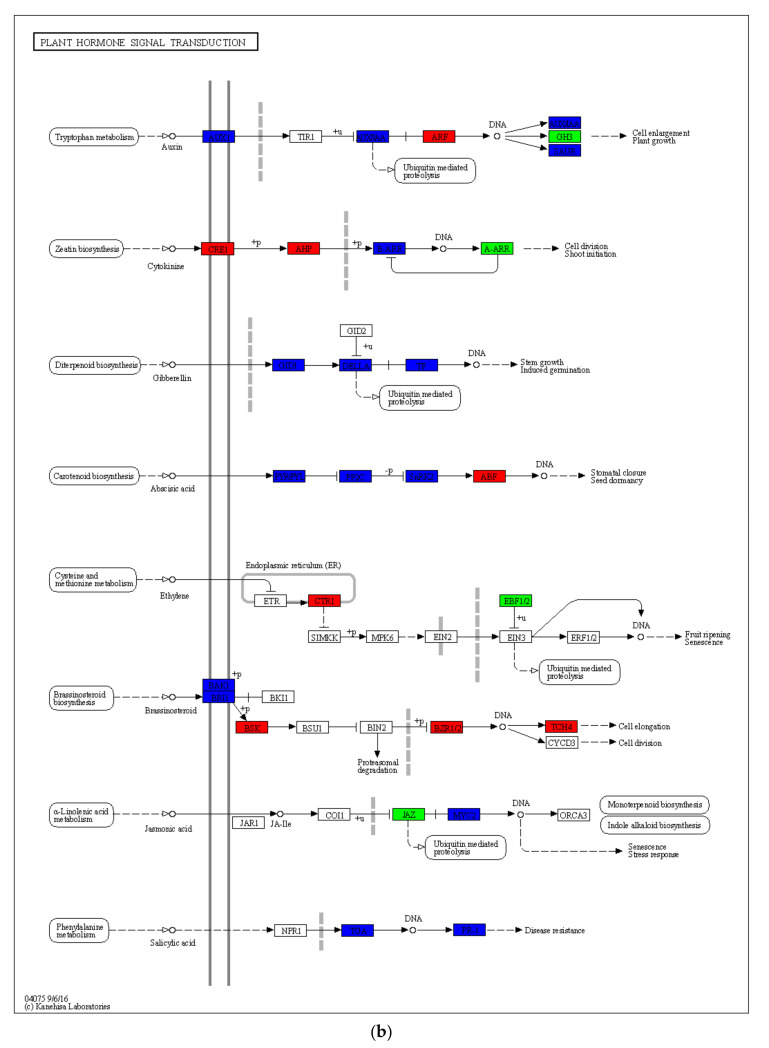
Graph of gene expression differences in plant hormone signaling pathways. (**a**) HN65 and (**b**) HN44.

In HN65 cells, 331 genes were involved in this pathway (Figure 5). In the auxin metabolic pathway, all genes encoding auxin influx carrier (AUX1) were downregulated, most of its downstream genes encoding auxin-responsive protein IAA were also downregulated, and most of the genes encoding GH3 and SAUR were downregulated, which also led to cell elongation and delayed plant development. In cytokinin metabolism, genes encoding CRE1, AHP, and A-ARR were upregulated to promote cell division (mainly shoot germination). In gibberellin metabolism, the number of downregulated genes was greater than that of upregulated genes encoding GID1, DELLA, and phytochrome-interacting factor 4, which may lead to the arrest of stem growth. Under the stimulation of abscisic acid signal, the activities of some downstream proteases changed significantly. First, three of the nine genes encoding the abscisic acid receptor PYR/PYL family were upregulated (*LOC100810273*, *LOC100813825*, and *LOC100783267*), second, all nine genes encoding protein phosphatase 2C were upregulated, and one of the two genes encoding SNRK2 was upregulated (*LOC100782714*), most genes encoding ABA-responsive element binding factor (ABF) were upregulated (five upregulated, two downregulated) to promote stomatal closure. In the ethylene metabolism, owing to the downregulation of a large number of genes in the MAKP family, the downstream genes encoding EIN3-binding F-box protein and ethylene-responsive transcription factor 1 were all downregulated, resulting in the inhibition of ethylene metabolism. In the metabolism of brassinolide, there were a large number of genes encoding BAK1 and BRI1, and a large number of them were downregulated, which also led to their downstream BRI1 kinase inhibitor 1, BR-signaling kinase, brassinosteroid-resistant 1/2, and xyloglucan:xyloglucosyl transferase, which inhibit the expression of TCH4 and cyclin D3, ultimately inhibiting cell division and elongation. Jasmonic acid was produced during the metabolism of α-linolenic acid. In jasmonic acid metabolism, the gene *LOC100792200* in jasmonic acid-amino synthetase was upregulated, and the number of upregulated genes in the downstream genes encoding transcription factor MYC2 was also greater than that of downregulated genes, which may promote plant response to drought stress. In salicylic acid metabolism, which was also affected by the MAPK pathway, PR1 expression was inhibited, and plant disease resistance was weakened. In HN44 cells, 178 genes were involved in this pathway. In the auxin metabolism pathway, the number of genes involved is much less than that of HN65, and two of the three genes encoding AUX1 were upregulated (*LOC100792625*, *GMLAX15*), all the genes encoding ARF were upregulated, and part of the genes encoding AUX/IAA and SUAR were upregulated, trying to ensure the growth of plants under adversity. In cytokinin metabolism, unlike HN65, the gene encoding A-ARR was downregulated, but only one gene was involved, *LOC100778707*, which may have an inhibitory effect on cell division or bud growth. In gibberellin metabolism, HN44 and HN65 were not very different, but the number of genes involved was lower than that of HN65. Under stimulation by abscisic acid signal, two out of eight genes encoding the abscisic acid receptor PYR/PYL family were upregulated (*LOC100499973* and *LOC100783267*), followed by eight out of ten genes encoding protein phosphatase 2C, and two genes encoding SNRK2 were upregulated. One of the genes was upregulated (*LOC100782714*) and all four genes encoding ABA-responsive element binding factor (ABF) were upregulated (*BZIP10*, *BZIP71*, *GMABI5*, and *LOC100819313*) to promote stomatal closure. The difference in the metabolism of brassinolide between the two varieties was large. In HN44, the number of genes encoding BRI1 was upregulated more than downregulated, and the expressions of BSK, BZR1/2, and TCH4, which were inhibited in HN65, were all upregulated in HN44. Among them, the two genes encoding TCH4 showed opposite expression patterns in the two varieties. The log_2_FC of *LOC100800547* and *LOC100785313* were 2.75 and 2.14 in HN44, and −6.57 and −5.58 in HN65, respectively. One of the causes for the difference in drought resistance between the two cultivars is the differing expression patterns of these two genes, which resulted in active cell elongation under stress in HN44 but inhibited in HN65. In jasmonic acid metabolism, only one of the 10 genes encoding MYC2 was downregulated, while 6 of the 13 genes involved in HN65 were downregulated, indicating that the response to stress was stronger in HN44 than in HN65. In salicylic acid metabolism, the upregulated expression of the gene *GMBZIP19* encoding the transcription factor TGA and the gene *LOC100790142* encoding PR1 may confer disease resistance to plants. In general, the metabolism of various plant hormones in HN65 was inhibited to a greater extent, and the overall performance of HN44 was stronger than that of HN65, particularly in the metabolic pathway of brassinolide.

Taken together, these results suggest that plants resist drought stress in a variety of ways. At the transcriptional level, it mainly involves the TCA cycle, glycolysis/gluconeogenesis, pentose phosphate pathway, plant hormone metabolism, synthesis of secondary substances, amino acid metabolism, sugar metabolism, and photosynthesis. In addition, both varieties assist in resistance to drought stress through a number of unique pathways. HN65 mainly includes monoterpenoid biosynthesis, phosphonate and phosphinate metabolism, biosynthesis of various secondary metabolites, and synthesis and degradation of ketone bodies. HN44 mainly includes indole alkaloid biosynthesis, sulfur relay system, autophagy-other, valine, leucine, and isoleucine biosynthesis, and glycosylphosphatidylinositol (GPI)-anchor biosynthesis.

### 2.6. PCA and OPLS-DA Analysis

Through principal component analysis (PCA), the overall metabolic differences among the HN65CK, HN65S0, HN44CK, and HN44S0 groups were preliminarily understood (Supplementary Figure S1). For HN65, the PC1 score was 44.36%, and the first two principal components accounted for 67.78% of the total variance. For HN44, the PC1 score was 44.25%, and the first two principal components accounted for 63.16% of the total variance. In each cultivar, there was a clear distinction between the treatment and control groups, indicating that drought stress had an impact on the metabolic levels of the two cultivars.

**Figure 6:**
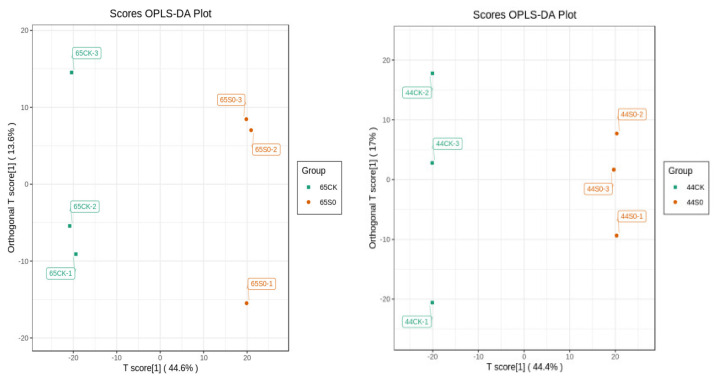
OPLS−DA score map. (**left**) HN65, (**right**) HN44.

Differential metabolites were identified using orthogonal partial least squares discriminant analysis (OPLS-DA). The OPLS-DA score map was used to show the differences between various groups (Figure 6). For HN65, the R2Y, R2X, and Q2 scores were 0.583, 0.999, and 0.918, respectively, and for HN44, the R2Y, R2X, and Q2 scores were 0.613, 1, and 0.911, respectively. Q2 was greater than 0.9 in both varieties, and the OPLS-DA model was more stable, indicating that drought stress affected metabolite levels. Additionally, 200 random permutations and combined alignment tests were used to validate the OPLS-DA model (Supplementary Figure S2).

### 2.7. Identification and Classification of Differential Metabolites in Response to Drought in Two Varieties

**Figure 7:**
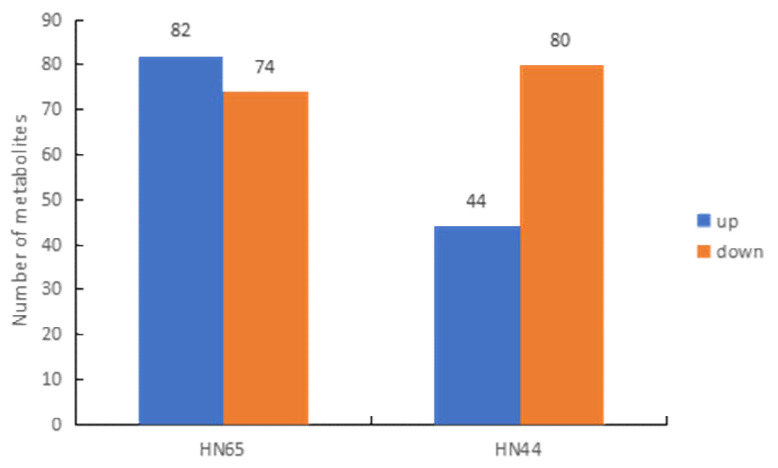
Quantitative statistics of differential metabolites.

Differential metabolites were identified using a combination of the VIP and FC values (Figure 7). A total of 156 differential metabolites were found in the HN65 treatment after drought stress (Supplementary Table S4), with 74 downregulated and 82 upregulated, according to the primary classification of substances, including 16 amino acids and their derivatives, 18 phenolic acids, 17 nucleotides and their derivatives, 45 flavonoids, 5 lignins and coumarins, 24 other substances (sugars, alcohols, and vitamins), 3 alkaloids, 1 terpenoid, 13 organic acids, and 14 lipids. In the HN44 treatment after drought stress, a total of 124 differential metabolites were found (Supplementary Table S5), of which 80 were downregulated and 44 were upregulated, including 8 amino acids and their derivatives, 8 phenolic acids, 6 nuclear glycosides and their derivatives, 49 flavonoids, 2 lignins and coumarins, 19 other substances (mainly sugars, alcohols, and vitamins), 4 alkaloids, 2 terpenes, 12 organic acids, and 14 lipids.

### 2.8. Metabolism Differences of Flavonoids

Flavonoids accounted for the largest proportion of differential metabolites in both varieties. Among the 45 flavonoids in HN65, there were 20 isoflavones (19 upregulated and 1 downregulated), 3 other flavonoids, 1 flavanol, 6 flavonols, 6 flavonoids, 1 dihydroflavonol, 7 dihydroflavones, and 1 chalcone. Of these, only 8 were downregulated and 37 were upregulated. Interestingly, the opposite trend was observed in HN44, where only 7 of the 49 flavonoids were upregulated and 42 were downregulated. This included 21 isoflavones (all downregulated), 1 flavanol, 8 flavonols, 11 flavonoids, 3 dihydroflavonoids, 2 dihydroflavonols, and 3 chalcones. Thirteen flavonoid metabolites were detected in both varieties, but their expression was mostly the opposite: afrormosin, 5-Hydroxy-6,7-dimethoxyflavone, pterocarpine, aracarpene 1, gancaonin N, ononin, 6″-O-Acetyldaidzin, and 6″-O-Malonyldaidzin had opposite expression patterns between the two cultivars, upregulated in HN65 and downregulated in HN44. Chrysoeriol-7-O-(6″-acetyl)glucoside was downregulated in both cultivars, and Monohydroxy-trimethoxyflavone-O-(6″-malonyl)glucoside was upregulated in both cultivars. Flavonoids scavenge ROS. The reason for this may be that HN44 maintains the ROS level in a dynamic equilibrium state through its own antioxidant system, thereby reducing the biosynthesis of flavonoids and energy consumption, which is used to maintain plants under drought conditions. Pathways to normal development, such as increased alpha-ketoglutarate levels, are marked by an enhanced TCA cycle. The drought resistance of HN65 is weak, and it is being attacked by a large amount of ROS at this time; therefore, high expression of flavonoids is required to clear ROS in time. In addition, low expression of flavonoids can promote the transport of auxin and ensure development under adverse conditions. Therefore, HN44 was more adaptable to drought conditions.

### 2.9. Analysis of the Overall Changes in the KEGG Metabolic Pathway

**Figure 8:**
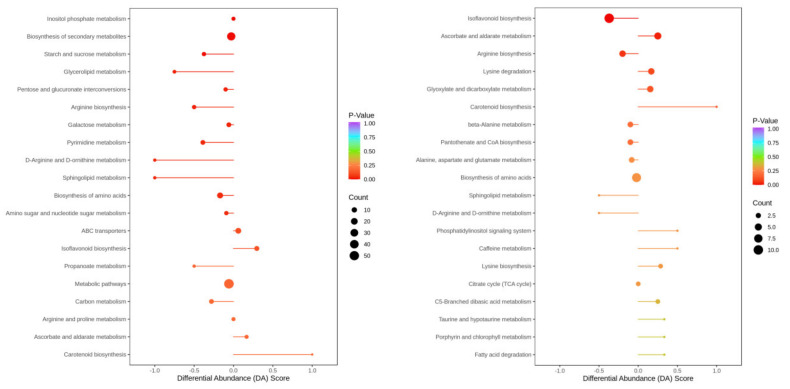
Differential abundance score map. (**Left**) HN65; (**Right**) HN44.

The differential abundance score (DA score) is a metabolic change analysis tool based on pathways, and it can capture the total changes in all differential metabolites in a pathway (Figure 8). In HN65, according to the P value, the top five pathways for differential metabolite enrichment were inositol phosphate metabolism, biosynthesis of secondary metabolites, starch and sucrose metabolism, glycerolipid metabolism, and arginine biosynthesis. The expression of some pathways tended to be downregulated, and only the expression of isoflavonoid and carotenoid biosynthesis pathways tended to be upregulated. In HN44, the first five pathways enriched for differential metabolism were isoflavonoid biosynthesis, ascorbate and aldarate, arginine biosynthesis, lysine degradation, glyoxylate, and dicarboxylate. Among the top 20 pathways, only 7 were downregulated. Overall, more pathways appeared to be prone to downregulation in HN65, suggesting that drought may have caused more damage to HN65.

### 2.10. Combined Analysis of Transcriptome and Metabolome

**Figure 9:**
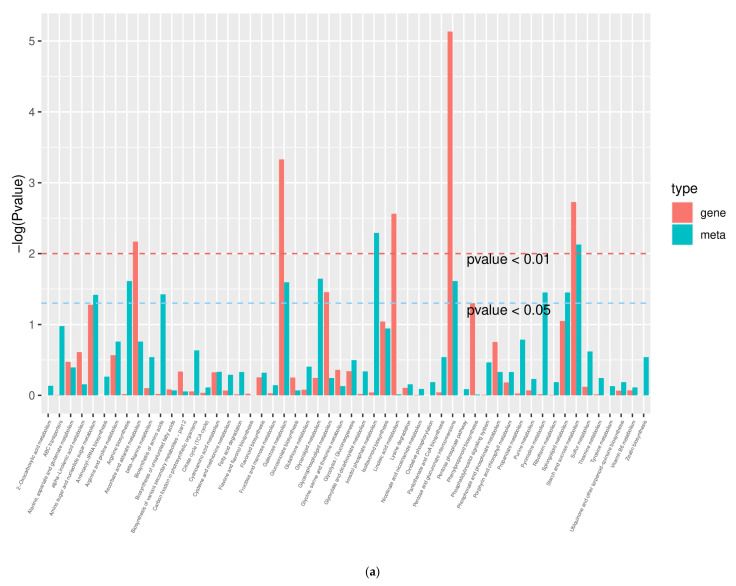

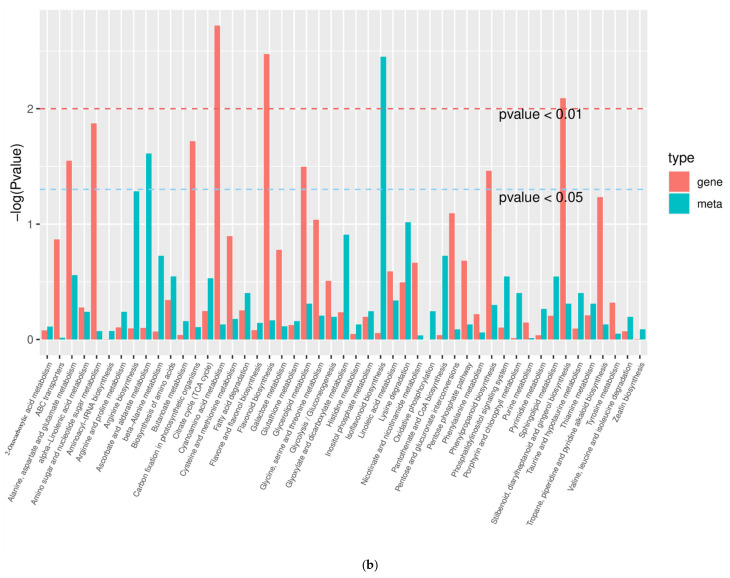
KEGG enrichment analysis P value histogram. The abscissa represents the metabolic pathway, the red in the ordinate represents the enrichment *p* value of differential metabolites, and the green represents the enrichment *p* value of the differential gene, which is represented by -log (*p* value), the higher the ordinate, the higher the degree of enrichment. (**a**) HN65; (**b**) HN44.

Differential genes and metabolites were simultaneously subjected to KEGG analysis to understand the pathway changes that were jointly involved in the response to drought (Figure 9). HN65, DEGs and DAMs were enriched in 54 pathways, and the top 5 pathways with the highest enrichment were inositol phosphate metabolism, starch and sucrose metabolism, glycerolipid metabolism, pentose and glucuronate interconversion, and arginine biosynthesis. HN44, DEGs, and DAMs were enriched in 49 pathways, and the top 5 pathways with the highest enrichment were isoflavonoid biosynthesis, ascorbate and aldarate metabolism, arginine biosynthesis, lysine degradation, glyoxylate metabolism, and dicarboxylate metabolism. In addition, the TCA cycle, pentose phosphate pathway, and glycolysis/gluconeogenesis pathway were enriched. Overall, the enrichment of HN44 was relatively high. Based on the results of transcriptomics and metabolomics, we further analyzed the changes in the differential genes and metabolites involved in isoflavone biosynthesis and the TCA cycle to better understand their mechanisms of response to drought.

### 2.11. Differences in the Expression of DEGs and DAMs in the Isoflavone Biosynthesis Pathway between the Two Varieties

**Figure 10:**
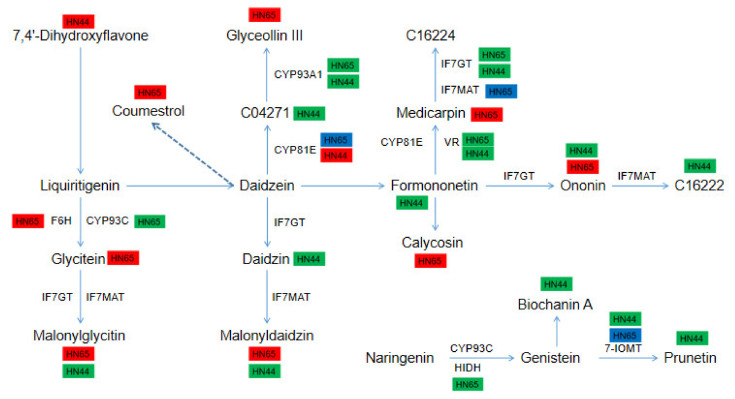
Map of gene and metabolite changes in isoflavone biosynthetic pathways. Upregulated genes or metabolites are represented in red, downregulated genes or metabolites are represented in green, and both upregulated and downregulated genes are represented in blue, the same below.

In HN65, 8 metabolites and 15 genes were involved in this pathway (Figure 10). Most of the genes were downregulated at the gene level. In addition to the upregulation of *CYP71D10* encoding F6H (flavonoid 6-hydroxylase), the genes encoding CYP93A1, HIDH, CYP93C, IF7GT, and VR were all downregulated, and the genes encoding CYP81E, IF7MAT, and 7-IOMT were partially downregulated. However, at the metabolic level, all eight metabolites were upregulated, including Coumestrol, Glycitein, Malonylglycitin, Ononin, Calycosin, Medicarpin, Glyceollin III, and Malonyldaidzin, which may be due to a negative feedback regulation response (from metabolites to gene). In HN44, 10 metabolites and 6 genes were involved in this pathway. All genes and metabolites were downregulated, except for the gene *LOC100784120* encoding CYP81E, which was upregulated. The major metabolites included 7,4’-Dihydroxyflavone, 3,9-Dihydroxypterocarpan, Formononetin, Ononin, Formononetin 7-O-glucoside-6″-O-malonate, Daidzin, Malonyldaidzin, Malonylglycitin, Prunetin, and Biochanin A. Among them, Malonyldaidzin and Malonylglycitin accumulated in the two cultivars, but their expression patterns were opposite.

### 2.12. Differences in the Expression of DEGs and DAMs in the TCA Cycle between the Two Varieties

**Figure 11:**
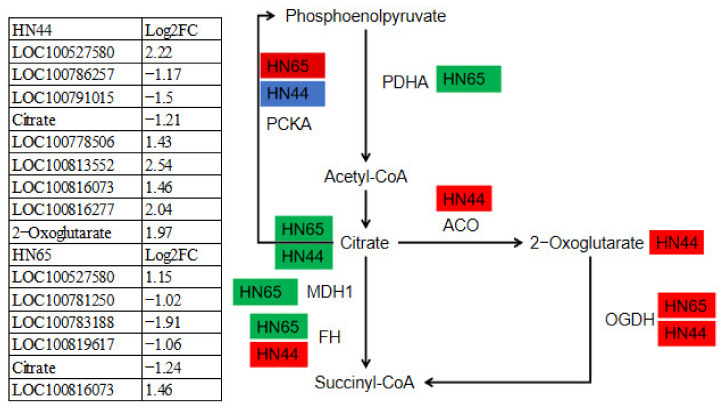
Diagram of gene and metabolite changes in the TCA cycle.

Plant resistance is influenced by the tricarboxylic acid cycle. Understanding the changes in gene and metabolite expression in the TCA cycle of the two varieties may explain some causes for the drought resistance discrepancies between the two varieties (Figure 11). Five genes and one metabolite were involved in HN65. The expression of *LOC100527580*, the gene encoding PCKA, was upregulated, which may promote the production of phosphoenolpyruvate. The gene encoding PDHA, *LOC100781250*, was downregulated, which inhibited the conversion of pyruvate to acetyl-CoA, thereby affecting the production of citrate, which is one of the reasons why citrate was downregulated in HN65. In addition, the gene encoding MDH1, *LOC100783188*, was also downregulated. The FH-encoding gene *LOC100819617* was downregulated, which inhibited the conversion of malate to oxaloacetate. To compensate for this process, the OGDH-encoding gene, *LOC100816073*, was upregulated, which may promote succinyl-CoA synthesis. In HN44, there are seven genes and two metabolites involved in this pathway, among the three genes encoding PCKA, one was upregulated (*LOC100527580*), two were downregulated (*LOC100786257*; *LOC100791015*), and the expression level of citrate was also downregulated. Genes encoding FH and OGDH were upregulated. In addition, the genes encoding ACO were also upregulated, which led to the accumulation of 2-Oxoglutarate in HN44, 2-Oxoglutarate was involved in multiple pathways including glyoxylate and dicarboxylic acid metabolism, metabolic pathways, biosynthesis of secondary metabolites, carbon metabolism, 2-oxocarboxylic acid metabolism, and amino acid biosynthesis. In general, HN65 is inhibited in many pathways in the TCA cycle, whereas HN44 is more active in the TCA cycle under drought stress, and the accumulation of 2-Oxoglutarate activates multiple droughtresistance pathways, which may explain why HN44 has stronger drought resistance than HN65. 

### 2.13. Comprehensive Understanding of Plant Drought Resistance Pathways

**Figure 12:**
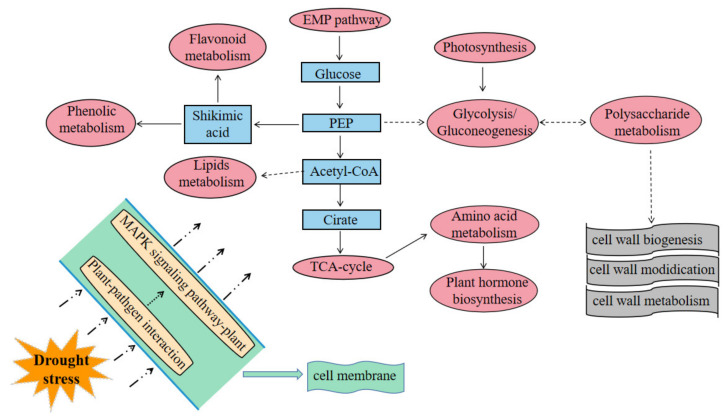
Schematic diagram of soybean response to drought stress.

Based on the literature and our omics data, we proposed a model for the soybean response to drought stress (Figure 12). When subjected to drought stress, the MAPK signaling pathway is triggered by sensors on the cell membrane such as cell surface pattern recognition receptors (PRR), thereby activating the defense genes of antimicrobial compounds. In addition, the TCA cycle, EMP pathway, and glycolysis-process-based (both in terms of transcription and metabolism) activation of drought-resistant pathways, including photosynthesis, hormone metabolism, amino acid synthesis, phenol and flavonoid metabolism, and lipid metabolism. Outside the cell membrane, it manifests as cell wall synthesis and modification. According to the differences between varieties, there are some unique drought resistance pathways within the varieties, such as sulfur, vitamin B6, and butanoate metabolism.

## 3. Discussion

Drought stress is one of the most damaging abiotic stresses to plant development and productivity and poses a threat to the long-term viability of agricultural production [21]. In a study of *Paeonia lactiflora* in response to drought stress, the ROS system, chlorophyll degradation and photosynthetic capacity, biosynthesis, and sugar metabolism were significantly affected [22]. In our study, photosynthesis was inhibited under drought stress, and carbohydrate metabolism was enhanced to a certain extent to maintain growth and development. Zhao et al. [23] used transcriptomic and metabolomic analyses to reveal key metabolites, pathways, and candidate genes in *Sophora davidii* (Franch) keel seedlings under drought stress. They found that L-aspartic acid and L-phenylalanine were involved in multiple metabolic pathways, and they also identified some genes that respond to drought, mainly belonging to the WRKY, MYB family. Although some amino acids such as L-Arginine and L-Citrulline were also found in our study, most of the main metabolites were flavonoids and the differential genes were also mainly involved in the synthesis of flavonoids, hormone metabolism, and other pathways. This suggests that different species have many similar responses to drought but differ in their core drought resistance pathways.

Abiotic stress defense responses are modulated by several plant hormones [24]. Under drought conditions, the synthesis (or metabolism) of related hormones, including zeatin, abscisic acid, and brassinolide, is activated in both varieties. At the metabolic level, abscisic acid accumulated in both the varieties. The log_2_FC was 2.96 in HN65 and 3.01 in HN44. At the transcriptional level, ABF binded to ABRE and led to the upregulation of ABA-responsive genes; all four genes encoding ABF were upregulated in HN44. Five genes were also upregulated in HN65 cells. Through comparative analysis, it was determined that two genes encode ABF in two soybean varieties (*BZIP71* and *LOC100819313*). Xuan et al. [25] found that ABA and SA signaling pathways were also involved in the response of two soybean varieties to drought stress. There was a significant difference between JD (drought-resistant) and N1 (drought-sensitive). Three and seven upregulated genes were found to encode ABF involved in ABA signaling in N1 and JD, respectively. ABA is closely related to MAPK signaling and the MAPK cascade has been shown to be related to ABA signaling [26]. The MAPK signaling pathway has recently been shown to play a key role in abiotic stress signaling [27]. After soybeans were subjected to drought stress, the MAPK pathway was activated in both varieties. In HN65 cells, although MKK1, MKK2, MPK4, MPK3, and MPK6 were activated, their expression was inhibited. MPK4 and MPK6 are rapidly activated under conditions of low temperature, low humidity, high osmotic pressure, tactile sensation, and damage [28]. Salt, drought, and trauma also activate MKK1, which phosphorylates MPK4 and is involved in abiotic stress signaling [29]. In HN44, only MKK1 and MKK2 were activated, but the overall inhibitory effect of HN65 was significantly greater than that of HN44, which may be related to two genes: *LOC100802571* and *LOC100814585*. These two genes were upregulated in HN44 and downregulated in HN65, and their encoded FLS2 is the “key” to activate the MAPK pathway [30]. Different expression patterns of FLS2 can affect the expression of the MAPK pathway, which in turn affects various metabolic pathways, including phytohormone signaling. These two genes could be used as candidate genes that affect plant drought tolerance.

Flavonoids are present in a wide range of healthy food products. The principal biological action of flavonoids is their antioxidant activity, which has been extensively investigated. By scavenging ROS, activating antioxidant enzymes, inhibiting oxidase, and decreasing tocopherol free radicals, flavonoids reduce free radical-induced damage [31]. The two cultivars accumulated substantial amounts of flavonoids during drought stress and each accounted for the largest proportion of the differential metabolites. In HN65, among the upregulated TOP10 metabolites, there were eight kinds of flavonoids, and in HN44, there was also some accumulation of flavonoids, but the upregulation was lower than that in HN65, and most of the flavonoid metabolites in HN44 were downregulated. In a drought study on mulberry, researchers discovered that flavonoids, organic acids, carbohydrates, and other compounds showed a dynamic pattern in response to drought stress, with their levels dropping dramatically [32]. Therefore, the accumulation or reduction of flavonoids under drought stress is a normal response in plants. There are many reasons for this, including the treatment period, species, and the degree of drought stress. In our previous study, which reported the metabolomic changes in these two cultivars under drought stress in the R3 phase, amino acids accounted for the largest proportion of the identified metabolites. Regarding flavonoids, four flavonoids accumulated in HN44 and seven flavonoids accumulated in HN65 [33]. Therefore, we believe that soybeans respond differently to drought stress at different stages. In the seedling stage, flavonoids may be the main metabolites for drought resistance, whereas in the R3 stage, amino acids are the most important for drought resistance. Under the same experimental treatment, the metabolic characteristics of the different periods varied significantly.

Additionally, drought led to systematic changes in a broad metabolic network. In the two soybean varieties, drought significantly affected metabolic pathways, including the TCA cycle and the glycolytic pathway. The expression of many genes was also significantly affected at the transcriptional level. The TCA cycle is the most significant central metabolic process, connecting nearly all individual metabolic pathways and the last common oxidation pathway of carbohydrates, fats, and amino acids [34]. Drought altered variables such as transamination, Krebs cycle, glycolysis, glutamate-mediated proline biosynthesis, shikimate-mediated secondary metabolism, and aminobutyric acid metabolism [35]. In this study, we focused on changes in genes and metabolites involved in the TCA cycle. We observed some differences in the accumulation of 2-Oxoglutarate between the two varieties. HN44 showed increased expression, but not in HN65. Because 2-Oxoglutarate can mediate many routes, it is possible that this is one of the causes of the differences in drought resistance between the two cultivars. Zhang et al. [36] created a metabolic model of licorice under drought stress in their study. TCA and glycolytic pathways were the most important components of drought tolerance. In addition, based on the transcriptome and metabolome data, we constructed a soybean drought resistance model, including gene metabolism and metabolite metabolism pathways. In this model, the TCA cycle, glycolysis, and pentose phosphate pathway were the internal metabolic basis, while the PRR located on the cell membrane can activate the MAPK pathway, triggering further signaling, such as phytohormone signaling. However, if we want to construct a comprehensive drought resistance network in a certain period, we may also need to understand the changes in proteomic and physiological aspects (antioxidative enzymes, osmotic regulators, etc.), which is one of our future studies.

## 4. Materials and Methods

### 4.1. Plant Material and Experimental Treatment

Two cultivars with certain differences in drought tolerance during seed germination were screened in the previous stage: Heinong 65 (HN65) and Heinong 44 (HN44) as test materials. Previous studies have shown that the drought resistance of HN65 is weaker than that of HN44 [10]. The experiment adopted a method combining river sand potted planting and PEG-6000 osmotic drought stress. Soybean seeds with full grain, consistent size, and no pests or diseases were selected for sowing; the seedlings were fixed after cultivation, and three seedlings with robust growth and consistent growth were kept in each pot. Each treatment was guaranteed to have more than three pots. The opposite true leaves were fully unfolded and drenched with distilled water once a day, 500 mL each time; when the opposite true leaves were fully unfolded, water Hoagland nutrient solution was added once a day, 500 mL each time. To prevent the accumulation of salt in the sand culture, the plants were rinsed with water every three days, 500 mL each time. When the seedlings reached the V3 stage, they were subjected to drought stress, and 500 mL of nutrient solution containing 15% PEG-6000 was poured every morning and evening for four days. The time to reach pretreatment was determined by sampling at 9:00–10:00 in the morning, and the sampling site was a mixture of the penultimate leaf and the penultimate third leaf. Each treatment included a minimum of three biological replicates. Each treatment was marked separately, that is, the control group was HN65CK and HN44CK, and the drought treatment was marked as HN65S0 and HN44S0. The subsequent analysis based on the analysis between groups, that is, HN65 represents HN65CK vs. HN65S0, HN44 represents HN44CK vs. HN44S0.

### 4.2. Transcriptomic Assay Methods

The cDNA libraries were sequenced on the Illumina sequencing platform by Metware Biotechnology Co., Ltd. (Wuhan, China). RNA degradation and contamination was monitored on 1% agarose gels. RNA purity was checked using the NanoPhotometer^®^ spectrophotometer (IMPLEN, CA, USA). RNA concentration was measured using Qubit^®^ RNA Assay Kit in Qubit^®^ 2.0 Flurometer (Life Technologies, CA, USA). RNA integrity was assessed using the RNA Nano 6000 Assay Kit of the Bioanalyzer 2100 system (Agilent Technologies, Santa Clara, CA, USA).

A total amount of 1 µg RNA per sample was used as input material for the RNA sample preparations. Sequencing libraries were generated using NEBNext^®^ UltraTM RNA Library Prep Kit for Illumina^®^ (NEB, USA) following the manufacturer’s recommendations and index codes were added to attribute sequences to each sample. Briefly, mRNA was purified from total RNA using poly-T oligo attached magnetic beads. Fragmentation was carried out using divalent cations under elevated temperature in NEBNext First Strand Synthesis Reaction Buffer(5X). First strand cDNA was synthesized using random hexamer primer and M-MuLV Reverse Transcriptase (RNase H-). Second strand cDNA synthesis was subsequently performed using DNA Polymerase I and RNase H. Remaining overhangs were converted into blunt ends via exonuclease/polymerase activities. After the adenylation of 3′ ends of DNA fragments, NEBNext Adaptor with hairpin loop structure was ligated to prepare for hybridization. In order to select cDNA fragments of preferentially 250~300 bp in length, the library fragments were purified with AMPure XP system (Beckman Coulter, Beverly, CA, USA). Then, 3 µL USER Enzyme (NEB, USA) was used with size-selected, adaptor-ligated cDNA at 37 °C for 15 min followed by 5 min at 95 °C before PCR. Then, PCR was performed with Phusion High-Fidelity DNA polymerase, Universal PCR primers, and Index (X) Primer. Finally, PCR products were purified (AMPure XP system) and library quality was assessed on the Agilent Bioanalyzer 2100 system.

The clustering of the index-coded samples was performed on a cBot Cluster Generation System using TruSeq PE Cluster Kit v3-cBot-HS (Illumia) according to the manufacturer’s instructions. After cluster generation, the library preparations were sequenced on an Illumina Hiseq platform and 125 bp/150 bp paired-end reads were generated. Use fastp v 0.19.3 to filter the original data, mainly to remove reads with adapters; when the N content in any sequencing reads exceeds 10% of the base number of the reads, remove the paired reads. When the number of low-quality (Q ≤ 20) bases contained in the reads exceeds 50% of the bases of the reads, this paired read will be removed. All subsequent analyses are based on clean reads. Use HISAT v2.1.0 to construct the index and compare clean reads to the reference genome. DESeq2 v1.22.1 was used to analyze the differential expression between the two groups, and the P value was corrected using the Benjamini and Hochberg method. The corrected P value and |log_2_foldchange| are used as the threshold for significant difference expression. The screening conditions for differential genes were |log_2_Fold change | ≥ 1 and FDR 0.05. Analysis of differentially expressed genes was performed using the Kyoto Encyclopedia of Genes and Genomes (KEGG, https://www.genome.jp/kegg, accessed on 1 April 2022) and pathway annotation. The Gene Ontology database was used to classify the genes according to their functions.

### 4.3. Metabolomics Assay Methods

Metware Biotechnology Co., Ltd. (www.metware.cn, accessed on 1 April 2022) (Wuhan, China) followed the conventional methods for sample preparation, extract analysis, metabolite identification, and quantification. Biological samples are freeze-dried by vacuum freeze-dryer (Scientz-100F). The freeze-dried sample was crushed using a mixer mill (MM 400, Retsch, Shanghai, China) with a zirconia bead for 1.5 min at 30 Hz. Dissolve 100 mg of lyophilized powder with 1.2 mL 70% methanol solution, vortex for 30 s every 30 min for 6 times in total, place the sample in a refrigerator at 4 °C overnight. Following centrifugation at 12,000 rpm for 10 min, the extracts were filtrated (SCAA-104, 0.22 μm pore size; ANPEL, Shanghai, China, http://www.anpel.com.cn/, accessed on 1 April 2022) before UPLC-MS/MS analysis. 

The sample extracts were analyzed using an UPLC-ESI-MS/MS system (UPLC, SHIMADZU Nexera X2, https://www.shimadzu.com.cn/; MS, Applied Biosystems 4500 Q TRAP, https://www.thermofisher.cn/cn/zh/home/brands/applied-biosystems.html). The analytical conditions were as follows, UPLC: column, Agilent SB-C18 (1.8 µm, 2.1 mm × 100 mm); The mobile phase consisted of solvent A, pure water with 0.1% formic acid, and solvent B, acetonitrile with 0.1% formic acid. Sample measurements were performed with a gradient program that employed the starting conditions of 95% A, 5% B. Within 9 min, a linear gradient to 5% A, 95% B was programmed, and a composition of 5% A, 95% B was kept for 1 min. Subsequently, a composition of 95% A, 5.0% B was adjusted within 1.1 min and kept for 2.9 min. The flow velocity was set as 0.35 mL per minute. The column oven was set to 40 °C. The injection volume was 4 μL. The effluent was alternatively connected to an ESI-triple quadrupole-linear ion trap (QTRAP)-MS.

LIT and triple quadrupole (QQQ) scans were acquired on a triple quadrupole-linear ion trap mass spectrometer (Q TRAP), AB4500 Q TRAP UPLC/MS/MS System, equipped with an ESI turbo ion–spray interface, operating in positive and negative ion mode and controlled by Analyst 1.6.3 software (AB Sciex). The ESI source operation parameters were as follows: ion source; turbo spray; source temperature 550 °C; ion spray voltage (IS) 5500 V (positive ion mode)/−4500 V (negative ion mode); ion source gas I (GSI), gas II(GSII), and curtain gas (CUR) were set at 50, 60, and 25.0 psi, respectively; the collision-activated dissociation(CAD) was high. Instrument tuning and mass calibration were performed with 10 and 100 μmol/L polypropylene glycol solutions in QQQ and LIT modes, respectively. QQQ scans were acquired as MRM experiments with collision gas (nitrogen) set to medium. DP and CE for individual MRM transitions was carried out with further DP and CE optimization. A specific set of MRM transitions were monitored for each period according to the metabolites eluted within this period.

Differential metabolites were screened using a combination of fold change and VIP values; that is, metabolites with fold change ≥ 2 and fold change ≤ 0.5, and VIP ≥ 1. The differential metabolites were annotated using the KEGG database. R (base package) 3.5.1 was used for PCA analysis. MetaboAnalystR (R) 1.0.1 was used for OPLS-DA. Excel 2013 was used to generate this form.

## 5. Conclusions

In conclusion, we performed transcriptomic and metabolomic analyses to better understand the molecular mechanisms of soybean response to drought at the seedling stage. Under drought stress, soybeans maintain plant growth in various ways, including reducing photosynthesis and changing the cell wall structure. The MAPK signaling pathway plays an important role in plant drought resistance by mediating various metabolic pathways. Drought also alters the biosynthesis and signal transduction of other plant hormones, including auxin, gibberellin, brassinolide, and abscisic acid, and promotes substantial alterations (accumulation or reduction) in flavonoids. In the soybean drought resistance pathway, the TCA cycle is key to mediating processes, including hormone transport and amino acid synthesis. Therefore, in the process of crop production, the drought resistance of plants can be improved by spraying exogenous hormones or gene editing technology, so as to better cope with drought stress.

## Data Availability

Raw data have been deposited in the NCBI Short Read Archive (SRA) database, and the accession numbers are PRJNA823397 and PRJNA834849.

## Supplementary Materials

The following supporting information can be downloaded at: https://www.mdpi.com/article/10.3390/ijms23126869/s1.
