# Supplementary figures and images for: Transcriptomic and Metabolomic Analysis of Seedling-Stage Soybean Responses to PEG-Simulated Drought Stress
Source: Int J Mol Sci. 2022 Jun 20;23(12):6869. doi: 10.3390/ijms23126869 (PMC9224651; doi:10.3390/ijms23126869)

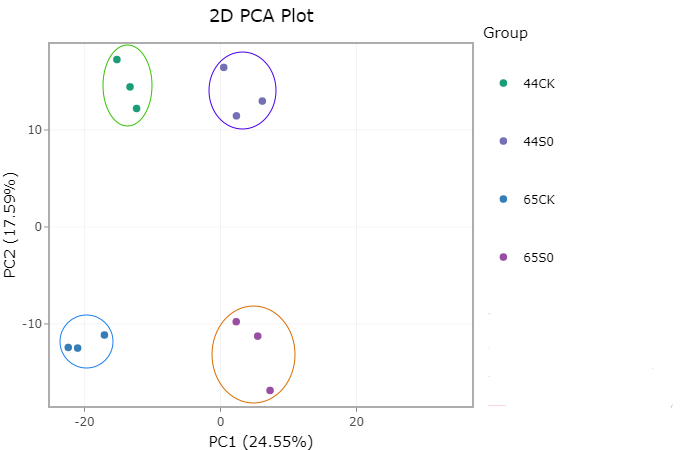

Supplement: Supplementary file 1 [file ijms-23-06869-s001.zip › Figure S1.png]

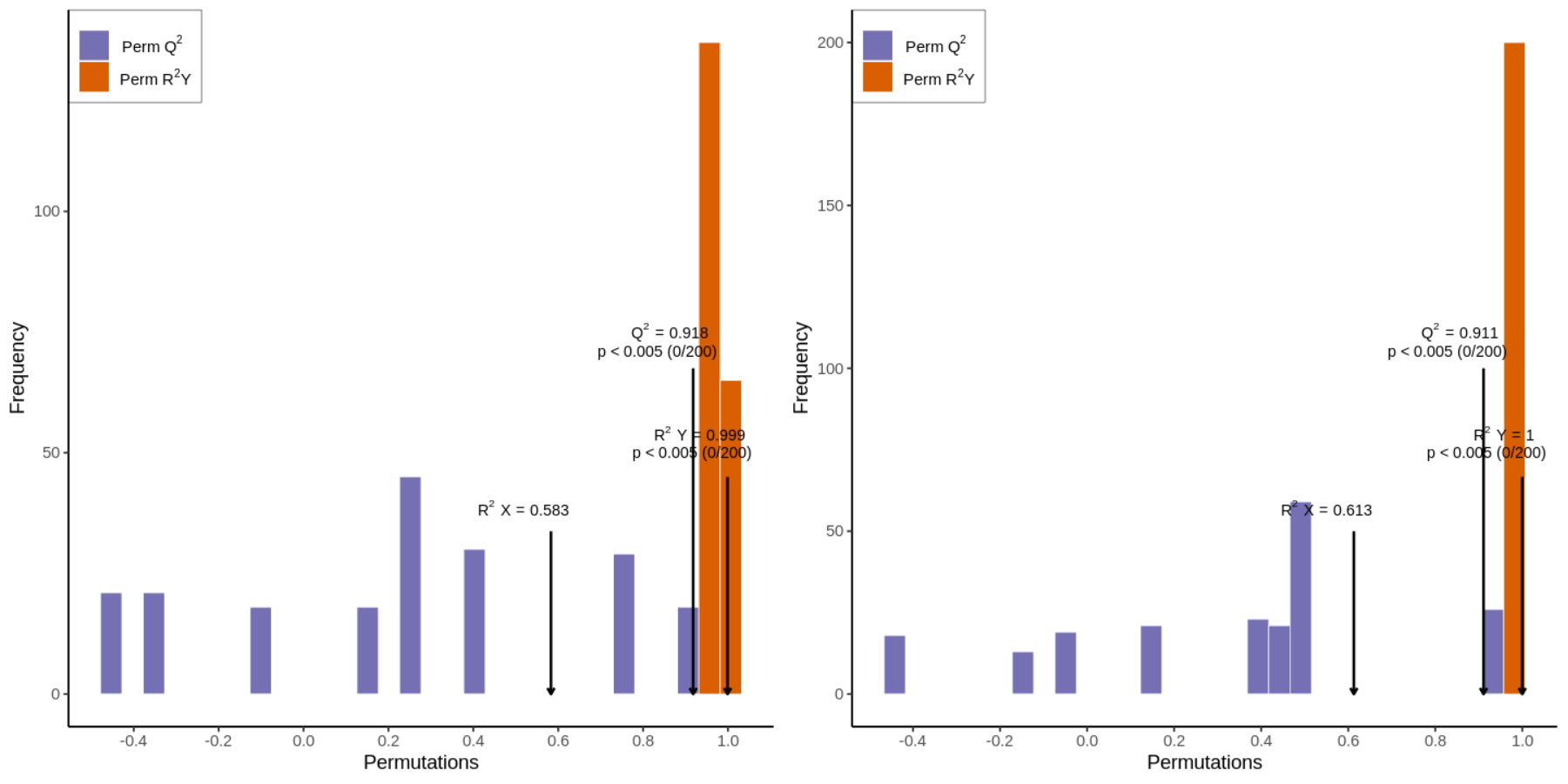

Supplement: Supplementary file 1 [file ijms-23-06869-s001.zip › Figure S2.tif]
